# The mediating role of coping in the relationship between perceived health and psychological wellbeing in recurrent urinary tract infection: the rUTI Illness Process Model

**DOI:** 10.1080/21642850.2024.2420806

**Published:** 2024-11-03

**Authors:** Abigail F. Newlands, Melissa L. Kramer, Kayleigh Maxwell, Jessica L. Price, Katherine A. Finlay

**Affiliations:** a School of Psychology and Clinical Language Sciences, University of Reading, Reading, UK; b Live UTI Free Ltd, Dublin, Ireland; c Department of Psychology, Faculty of Natural Sciences, University of Stirling, Stirling, UK

**Keywords:** Urinary tract infection, chronic illness, resilience, pain catastrophising, depression, anxiety, women’s health, structural equation modelling

## Abstract

**Background:** Recurrent urinary tract infection (rUTI) is associated with significant symptom and quality of life burden. Given the unique challenges in diagnostics and management, healthcare disillusionment and stigmatisation which distinguish rUTI from other urological conditions, specific identification of the key illness processes experienced by this patient population is required. This study aimed to identify the unique illness processes and perceptions that contribute to quality of life in rUTI, through perceived health status, psychological wellbeing, and coping.

**Methods:** An international sample of adults living with rUTI (*N = *389, 96.9% female) completed a cross-sectional survey comprising the following standardised questionnaires: the EuroQoL EQ-5D-5L, Patient Health Questionnaire 9 (PHQ-9), Generalised Anxiety Disorder 7 (GAD-7), Connor-Davidson Resilience Scale–10 (CD-RISC-10), Pain Catastrophising Scale (PCS). Sociodemographic characteristics were also assessed. Structural equation modelling was conducted to identify the underlying constructs which contributed to psychological wellbeing in rUTI, establishing the ‘rUTI Illness Process Model’.

**Results:** The positive relationship between ‘perceived health status’ and ‘psychological wellbeing’ was partially mediated by ‘rUTI coping’, after controlling for the impact of household income and age (*p *< .001). The model demonstrated a large effect size (*R*^2 ^= .81) and good local and global fit. Overall, rUTI coping skills, boosted by resilience and weakened by pain catastrophising, contribute to a significant proportion of the positive relationship between perceived health status and psychological wellbeing in rUTI. A uniquely vulnerable patient phenotype emerges from this new research, with patients who are younger and/or of lower socioeconomic status at greater risk of poorer rUTI health outcomes and psychological wellbeing, potentially requiring further support.

**Conclusions:** The rUTI Illness Process Model establishes the crucial need to clinically characterise the individualised illness perceptions and metacognitive strategies held by people living with rUTI, revealing that patient-centred interventions targeting illness perceptions and coping strategies require prioritisation to enhance patient outcomes and the patient experience of living with rUTI.

## Introduction

Urinary tract infection (UTI), annually affecting over 400 million people worldwide (Zeng et al., 2022), is associated with high symptom burden and negative consequences for quality of life (QoL) (Colgan et al., 2004; Medina & Castillo-Pino, 2019). Recurrent UTI (rUTI), experiencing two or more episodes within six months or three or more within a year (Bonkat et al., 2020), is even more limiting, with considerable negative psychosocial and economic implications for individuals and wider society (François et al., 2016; Maxwell et al., 2023; Naber et al., 2022; Wagenlehner et al., 2018). Psychological wellbeing is particularly problematic, with over 60% of people living with rUTI facing depression and/or anxiety (Renard et al., 2015), and almost 80% experiencing an impaired sex life (Ciani et al., 2013). Challenging physiological symptoms, alongside an ongoing fear of new or worsening symptoms, contribute to social anxiety and isolation, as well as withdrawal from the workplace and financial loss (Izett-Kay et al., 2022; Maxwell et al., 2023; Naber et al., 2022; Wagenlehner et al., 2018). The urgent need to strengthen patient-centred rUTI care is well-established, given high patient healthcare disillusionment and critical challenges within rUTI management related to insufficient education and awareness, misaligned clinician and patient perspectives, stigmatisation, and missed diagnosis (Brubaker et al., 2023; Gleicher et al., 2023; Haley & Luke, 2024; Harding et al., 2022; Hearn et al., 2018; Maxwell et al., 2023; Pickard et al., 2018; Sanyaolu et al., 2023; Sosland & Stewart, 2021). Diagnosis for rUTI typically relies on standard urine culture, yet recent microbiological research has demonstrated that up to 58% of UTIs are missed by this method, informing recommendations to prioritise engagement with the patient perspective (Brubaker et al., 2023; Haley & Luke, 2024; Harding et al., 2022; Pickard et al., 2018). These challenges are particularly problematic within the rUTI patient population compared with other urological conditions, given unique factors such as rising antimicrobial resistance and the reliance upon repeated antimicrobial treatment due to a paucity of non-antimicrobial or other interventions (Haley & Luke, 2024; Maxwell et al., 2023).

To improve patient-centred care, it is essential to identify the unique illness processes and perceptions that contribute to rUTI patient outcomes and QoL. Whilst it is known that high rUTI symptom severity is associated with lower psychological wellbeing, it is possible that coping mechanisms may play a role within this relationship, as they do in other chronic health conditions such as interstitial cystitis/bladder pain syndrome (IC/BPS) (Chng et al., 2023; Katz et al., 2017). Resilience and pain catastrophising may particularly affect the ability to cope and adjust to living with ill health (Ong et al., 2010; Ramírez-Maestre & Esteve, 2013; Ziadni et al., 2018). Whilst resilience explains an individual’s ability to adapt to and recover from significant change or adversity (Ramírez-Maestre & Esteve, 2013), pain catastrophising is a maladaptive cognitive–affective response that involves negative thinking about pain symptoms, and is associated with rumination, magnification, and helplessness (Sullivan et al., 1995).

Despite living with poor health, individuals with higher resilience have an improved ability to maintain a higher level of functioning and experience more positive emotions (Ramírez-Maestre & Esteve, 2013). They are less likely than those with lower resilience to exhibit pain catastrophising tendencies, and more able to rebound from negative thoughts about symptoms (Ong et al., 2010). Individuals with high pain catastrophising may magnify the threat value of their pain and symptoms, and ultimately exhibit poorer coping and QoL (Ziadni et al., 2018). These relationships have been demonstrated in related urological patient populations, including IC/BPS and chronic prostatitis/chronic pelvic pain syndrome (CP/CPPS) (Giannantoni et al., 2021; Hedelin, 2012; Katz et al., 2017; Naliboff et al., 2015; Soriano et al., 2021). However, resilience and pain catastrophising have not yet been explored within rUTI. Given the unique challenges in rUTI diagnostics and management, healthcare disillusionment and stigmatisation which distinguish rUTI from other urological conditions, specific identification of the key illness processes experienced by this patient population is required. By statistically validating the psychological illness processes in rUTI and identifying potential group differences in the patient experience, future clinical and psychosocial interventions can be more targeted and patient-centred, improving patient outcomes and the experience of living with this impactful condition.

Currently, psychosocial interventions for rUTI are scarce, with most limited to tools designed to support diagnostics and patient and/or clinician education (for example, Jones et al., 2020; Metwally et al., 2021). Whilst there are psychological interventions developed for those living with IC/BPS, such as programmes employing mindfulness-based techniques and symptom management skills (Kanter et al., 2016; Lee et al., 2018), there are no known psychological interventions specifically aimed at improving rUTI-related QoL and/or psychological illness processes. rUTI-specific interventions must be developed in order to address the unique challenges experienced by this patient population. This study therefore aimed to provide an understanding of the distinct illness processes involved in rUTI to inform the development of these much-needed interventions, using structural equation modelling (SEM) to characterise the inter-relationships between perceived health status, psychological wellbeing, and rUTI coping.

## Materials and methods

### Study design and hypotheses

**Figure 1.**
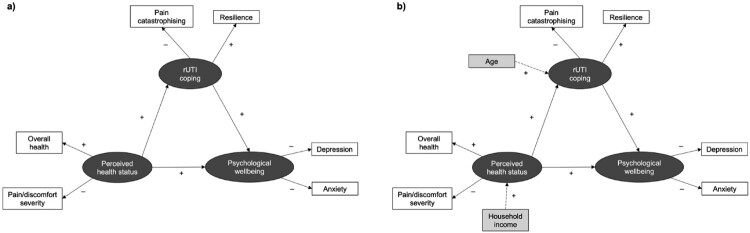
Hypothetical models tested with structural equation modelling (SEM) analysis. Figure 1a illustrates the initial, simplest model tested (in accordance with the parsimony principle), comprising three latent variables which are presented as dark grey oval shapes, and six observed variables which are presented as white rectangles. Figure 1b illustrates a full, more complex model, including an additional two observed variables (age and household income) as covariance variables, presented as light grey rectangles. The expected direction of each relationship is indicated with positive (+) and negative (−) signs. For example, it was hypothesised that greater pain catastrophising would be associated with lower rUTI coping (−), meanwhile greater resilience would be associated with greater rUTI coping (+). All relationships were expected to be statistically significant (*p* < .05).

An online, cross-sectional survey was conducted to collect data from a large, international patient sample for SEM analysis, testing explicit hypothetical models of how the following three latent variables interact in rUTI: ‘perceived health status’ (PHS), ‘rUTI coping’, and ‘psychological wellbeing’ (see Figure 1). The survey included five standardised questionnaires to assess these constructs, demonstrating strong psychometric properties (see Table 1). Data were collected between 17th August 2022 and 6th November 2022 using REDCap, a secure online survey and database tool. SEM is a robust statistical technique that facilitates exploratory and confirmatory modelling of multiple latent and observed variables simultaneously, thus is highly applicable to testing theoretical models in health outcomes research (Hays et al., 2005; Kline, 2016). Best practice recommendations for conducting and reporting SEM were followed throughout (Hays et al., 2005; Kline, 2016; Morrison et al., 2017; Schumacker & Lomax, 2010; Stone, 2021; Tabachnick & Fidell, 2012).

**Table 1.** Psychometric properties of standardised questionnaires.

Instrument	Construct measured	Items	Internal consistency (Cronbach’s α)	Test-retest reliability coefficient^a^	Convergent validity coefficient^b^	Sensitivity	Specificity
PHQ-9	Depression	9	.89	.84	.73	88% (score ≥ 10/27)	88% (score ≥ 10/27)
GAD-7	Generalised anxiety disorder	7	.89	.87	.93	89% (score ≥ 10/21)	82% (score ≥ 10/21)
EQ-5D-5L	Quality of life	6	.79	.92	.76	–	–
CD-RISC-10	Resilience	10	.93	.88	.68	–	–
PCS	Pain catastrophising	13	.89	.88	.59	–	–

Note: PHQ-9: Patient Health Questionnaire–9 (Kroenke et al., 2001). GAD-7: Generalised Anxiety Disorder–7 (Spitzer et al., 2006). EQ-5D-5L: EuroQol 5-dimension 5-level questionnaire (Herdman et al., 2011). CD-RISC-10: Connor Davidson Resilience Scale–10 (Campbell-Sills & Stein, 2007). PCS: Pain Catastrophising Scale (Sullivan et al., 1995).

Hyphens (–) indicate that no coefficient/data is available or applicable.

^a^ Test-retest reliability coefficients include: intraclass correlation coefficients (ICC), Pearson’s correlation coefficients, and Spearman’s rank coefficients. All can be interpreted as values between 0 and ±1, with values closer to ±1 indicating greater strength than values closer to 0.

^b^ Convergent validity (or concurrent validity, a type of construct validity) coefficients include: Pearson’s correlation coefficients, and Spearman’s rank coefficients. All can be interpreted as values between 0 and ±1, with values closer to ±1 indicating greater strength than values closer to 0.

Based on key literature and known relationships in related chronic urological and pain conditions (Giannantoni et al., 2021; Herdman et al., 2011; Katz et al., 2017; Kroenke et al., 2001; Mirowsky, 2017; Smith et al., 2013; Soriano et al., 2021; Spitzer et al., 2006), it was hypothesised that: (i) both PHS and rUTI coping would directly positively predict psychological wellbeing, and (ii) PHS would indirectly positively predict psychological wellbeing through its association with rUTI coping (see Figure 1a). PHS was expected to be positively predicted by overall health (EQ-5D-5L VAS score) and pain/discomfort severity (EQ-5D-5L pain/discomfort item) (Herdman et al., 2011). rUTI coping was expected to be positively predicted by resilience (CD-RISC-10 total score) and negatively predicted by pain catastrophising (PCS total score) (Giannantoni et al., 2021). Psychological wellbeing was expected to be negatively predicted by depression (PHQ-9 total score) and anxiety (GAD-7 total score) (Kroenke et al., 2001; Spitzer et al., 2006).

Additionally, two covariance relationships were hypothesised (see Figure 1b): (i) household income was expected to positively predict PHS (Mirowsky, 2017), and (ii) age was expected to positively predict rUTI coping (Smith et al., 2013). All standardised model path coefficients were expected to be statistically significant (*p *< .05). A total of three latent variables and eight observed variables were included across the hypothetical models.

### Participants and sampling

A heterogeneous sample of 389 adults experiencing rUTI were recruited, representative of the vast, diverse patient cohort (see Table 2 for demographic characteristics). Most participants (*n = *300, 84.8%) were recruited via email newsletters and social media posts by Live UTI Free, a leading UTI research and patient advocacy organisation with broad, international reach. Snowball sampling extended this reach (Noy, 2008), with additional recruitment channels including participants and clinicians sharing the study information via social media (*n = *37, 9.51%), UTI support groups (*n = *22, 5.66%), among others such as word of mouth (*n = *30, 7.71%).

**Table 2.** Participant demographic characteristics.

Characteristic	*n*	%
Biological sex		
Female	377	96.9
Male	12	3.08
Gender		
Female	374	96.1
Male	12	3.08
Non-binary	2	.51
Prefer not to say	1	.26
Country of residence		
United Kingdom	153	39.3
United States	147	37.8
Canada	26	6.68
Australia	8	2.06
Ireland	5	1.29
Greece	4	1.03
India	4	1.03
Spain	4	1.03
Other^a^	38	9.77
Ethnicity		
Asian (including Asian American and Asian British)	10	2.57
Black (including African, African American, Caribbean, Black British)	3	.77
Hispanic or Latino American	5	1.29
Mixed ethnicity or multiple ethnic groups	4	1.03
Native Hawaiian or other Pacific Islander	2	.51
White (including Caucasian, White British, White European)	340	87.4
Other ethnicity	4	1.03
Prefer not to say	21	5.40
Fluency in English		
Native or bilingual	337	86.6
Advanced or proficient	52	13.4
Relationship status		
Married or in a civil partnership	199	51.2
In a relationship (unmarried)	117	30.1
Single	44	11.3
Divorced	14	3.60
Widowed	6	1.54
Separated	5	1.29
Other	2	.51
Prefer not to say	2	.51
Highest level of education		
Some high school / secondary school	7	1.80
High school / secondary school	65	16.7
Bachelor’s degree or equivalent	168	43.2
Master’s degree or equivalent	104	26.7
Doctoral level training or equivalent	16	4.11
Other professional qualification(s)	22	5.66
Prefer not to say	7	1.80
Annual household income (GBP)		
No current income	12	3.08
£1 – £9,999	15	3.86
£10,000 – £24,999	37	9.51
£25,000 – £49,999	102	26.2
£50,000 – £74,999	48	12.4
£75,000 – £99,999	40	10.3
£100,000 or more	59	15.2
Prefer not to say	76	19.5

Note: *N *= 389.

^a^ Other countries where *n* ≤ 3 comprise the following 29 countries listed alphabetically: Angola, Argentina, Austria, The Bahamas, Belgium, Croatia, Czech Republic, Denmark, Finland, France, Germany, Iceland, Israel, Italy, Jersey, Malawi, Mexico, Netherlands, New Zealand, Nigeria, Norway, Romania, Serbia, Slovakia, South Africa, Sweden, Thailand, Turkey, and Ukraine.

Inclusion criteria comprised a minimum age of 18 years old, and meeting the diagnostic criteria for rUTI (≥2 UTIs within the past six months or ≥3 UTIs within the past year) (Bonkat et al., 2020). Exclusion criteria comprised a current diagnosis of IC/PBS. Given microbiological findings that up to 58% of infections are missed by standard urine culture and published recommendations to prioritise patient symptom reporting (Brubaker et al., 2023; Harding et al., 2022; Pickard et al., 2018), including participants based on self-report of symptoms was suitable and facilitated access to the larger, more heterogeneous sample required for SEM analysis. Based on a priori sample size calculations, a minimum sample of 256 participants would facilitate SEM with up to three latent variables and eight observed variables (see Figure 1), detecting a statistically significant medium effect size (Cohen’s *d *= .30, *p *< .05) with 80% statistical power (Kline, 2016; Soper, 2022). Thus, sampling adequacy was achieved.

Participants were aged between 18 and 87 years old (*M *= 45.5, *SD *= 17.1), and most reported female biological sex, as expected given UTI prevalence statistics (*n = *377, 96.9%; see Table 2) (Foxman, 2014; Zeng et al., 2022). Thirty-seven countries were sampled, with participants predominantly residing in the UK (*n = *153, 39.3%) and USA (*n = *147, 37.9%; see Supplementary Material 1 for all countries).

### Procedure

Ethical approval was received by the School of Psychology and Clinical Language Sciences Research Ethics Committee, University of Reading (project reference no.: 2022-115-KF). After reviewing a participant information sheet detailing the study procedure and ethical considerations, participants provided e-consent to take part. After completing a screening questionnaire, eligible participants proceeded to complete the PHQ-9, GAD-7, CD-RISC-10, PCS, and EQ-5D-5L (Table 1) (Campbell-Sills & Stein, 2007; Herdman et al., 2011; Kroenke et al., 2001; Spitzer et al., 2006; Sullivan et al., 1995). A debrief form signposted participants to UTI and mental health support resources.

### Data handling and statistical analysis

A full data handling and statistical analysis strategy following best practice recommendations is provided in Supplementary Material 2 (Hays et al., 2005; Kline, 2016; Morrison et al., 2017; Schumacker & Lomax, 2010; Stone, 2021; Tabachnick & Fidell, 2012). Adhering to the parsimony principle, the simplest model possible was specified as an initial model (Figure 1a) (Kline, 2016). A full, more complex model (Figure 1b) was subsequently specified to strengthen model fit, including household income and age as possible covariance variables (see hypotheses) (Mirowsky, 2017; Smith et al., 2013).

Evaluation of each model’s fit was based on Satorra-Bentler scaled Chi-square statistics, local and global fit testing, statistical power results, and model effect estimates. Given the sensitivity of the Chi-square exact-fit test to large sample sizes (*N *> 300), it is typical for final fit inferences to be made based on local fit testing, including examination of correlation and standardised residuals, and global approximate fit statistics (Kline, 2016; Stone, 2021).

It was specified a priori that the following thresholds would indicate good fit: scaled RMSEA below .08, with its entire 90% confidence interval below .10 (*p *> .05); SRMR below .08; CFI above .95; and PNFI greater than .50 (Kline, 2016). A minimum target of 80% power was defined a priori (Kline, 2016).

## Results

### Descriptive statistics

The final sample comprised 389 adults with rUTI (96.9% female, *n = *377; see Table 2 for demographic characteristics and Table 3 for descriptive statistics for observed variables). Approximately a third of participants (*n = *147, 37.8%) reported an annual household income above £50,000, and almost three-quarters (*n = *288, 74.0%) reported holding a bachelor’s degree or higher. Participants reported an average of 3.62 UTI episodes in the past six months (*SD *= 2.90) and 7.06 in the past year (*SD *= 5.91).

**Table 3.** Descriptive statistics observed for each instrument.

Measure	Construct	*M*	*SD*	Range
PHQ-9	Depression	9.90	6.86	0–27
GAD-7	Anxiety	15.2	6.00	0–21
CD-RISC-10	Resilience	24.6	8.09	0–40
PCS	Pain catastrophising	24.0	14.4	0–52
EQ-5D-5L: VAS	Overall health	57.3	22.0	0–97
EQ-5D-5L: pain/discomfort item	Pain/discomfort severity	2.45	1.05	1–5

Note: *N *= 389.

PHQ-9: Patient Health Questionnaire–9 (Kroenke et al., 2001). 9-item questionnaire using a 4-point Likert scale ranging from 0 to 3, with higher scores indicating greater severity. Maximum score range = 0–27.

GAD-7: Generalised Anxiety Disorder–7 (Spitzer et al., 2006). 7-item questionnaire using a 4-point Likert scale ranging from 0 to 3, with higher scores indicating greater severity. Maximum score range = 0–21.

CD-RISC-10: Connor Davidson Resilience Scale–10 (Campbell-Sills & Stein, 2007). 10-item questionnaire using a 5-point Likert scale ranging from 0 to 4, with higher scores indicating greater resilience. Maximum score range = 0–40.

PCS: Pain Catastrophising Scale (Sullivan et al., 1995). 13-item questionnaire utilising a 5-point Likert scale ranging from 0 to 4, with higher scores indicating greater pain catastrophising tendencies. Maximum score range = 0–52.

EQ-5D-5L: EuroQol 5-dimension 5-level questionnaire (Herdman et al., 2011). The visual analogue scale (VAS) utilises a maximum score range 0–100, with higher scores indicating better overall health. The pain/discomfort item utilises a 5-point Likert scale ranging from 1 to 5, with higher scores indicating lower quality of life.

Over three-quarters of participants (81.2%, *n = *316) indicated moderate or severe anxiety, scoring ≥10 on the GAD-7 (Spitzer et al., 2006). Almost half of participants (42.4%, *n = *165) indicated moderate or severe depression, scoring ≥10 on the PHQ-9 (Kroenke et al., 2001), with a quarter (26.7%, *n = *104) reporting thoughts of suicide or self-harm on at least ‘several days’ within the two weeks prior to participation. Approximately a third of participants (29.8%, *n = *116) reported lower than average overall health on the day of questionnaire completion, scoring <50 on the EQ-5D-5L VAS. Over a third (36.8%, *n = *143) indicated a clinically significant level of pain catastrophising, scoring ≥30 on the PCS (Sullivan et al., 1995). Participants demonstrated high heterogeneity, representing the diversity of this patient cohort, with questionnaire scores spanning the maximum possible range (Table 3).

Multiple linear regression analyses indicated group differences within wellbeing and rUTI coping based on household income, education, and age (see Table 4). Overall, lower levels of household income and education predicted higher levels of depression, anxiety, and pain catastrophising, as well as lower resilience and overall health. Younger adults typically indicated higher levels of anxiety and pain catastrophising.

**Table 4.** Group differences in observed variable scores.

Characteristic	Age	Biological sex ^a^	Household income	Education level	Relationship status ^b^
Depression (PHQ-9)					
* R*^2^_Adj_	−.00	.00	.05***	.05***	.00
* F*(1, 387)	.21	1.42	16.4	19.0	1.98
* β*	−.01	−2.40	−3.79	−3.81	−1.28
* *95% CI (LB, UB)	−.05,−.03	−6.35, 1.56	−5.62,−1.95	−5.53,−2.09	−3.06, .51
Anxiety (GAD-7)					
* R*^2^_Adj_	.02**	.01*	.03**	.01*	−.00
* F*(1, 387)	9.03	4.01	10.1	4.67	.11
* β*	−.05	−3.51	−2.62	−1.69	.26
* *95% CI (LB, UB)	−.09,−.02	−6.95,−.06	−4.24,−1.00	−3.24,−.15	−1.31, 1.82
Pain catastrophising (PCS)					
* R*^2^_Adj_	.06***	.01*	.02**	.02**	−.00
* F*(1, 387)	23.9	4.24	7.00	9.07	.25
* β*	−.21	−8.65	−5.24	−5.57	−.96
* *95% CI (LB, UB)	−.28,−.12	−16.9,−.39	−9.15,−1.33	−9.21,−1.93	−4.72, 2.80
Resilience (CD-RISC-10)					
* R*^2^_Adj_	.00	.00	.03***	.04***	−.00
* F*(1, 387)	2.92	2.47	11.2	17.3	.82
* β*	.04	3.72	3.64	4.32	−.97
* *95% CI (LB, UB)	−.01, .09	−.94, 8.37	1.50, 5.77	2.28, 6.36	−3.09, 1.14
Overall health (EQ-5D-5L VAS)					
* R*^2^_Adj_	−.00	−.00	.05***	.02**	.01*
* F*(1, 387)	.00	.05	18.1	7.57	6.39
* β*	.00	−1.46	13.1	7.89	7.33
* *95% CI (LB, UB)	−.13, .13	−14.2, 11.3	7.05, 19.2	2.25, 13.5	1.63, 13.0

Note: *N *= 389. PHQ-9: Patient Health Questionnaire–9 (Kroenke et al., 2001). GAD-7: Generalised Anxiety Disorder–7 (Spitzer et al., 2006). PCS: Pain Catastrophising Scale (Sullivan et al., 1995); CD-RISC-10: Connor Davidson Resilience Scale–10 (Campbell-Sills & Stein, 2007); EQ-5D-5L VAS: EuroQol 5-dimension 5-level questionnaire Visual Analogue Scale (Herdman et al., 2011); CI: confidence interval; LB: lower bound; UB: upper bound.

*p < .05; **p < .01; ***p < .001.

^a^ Biological sex was coded as 1 = female, and 2 = male. Therefore, a negative beta (*β*) value would indicate that males in the sample scored lower than females (e.g., see the relationship between biological sex and anxiety).

^b^ Relationship status was coded as 1 = single, and 2 = in a relationship (including, for example, married, in a civil partnership, co-habiting, dating). Therefore, a positive beta (*β*) value would indicate that participants in the sample who were in a relationship scored higher than participants who were single (e.g., see the relationship between overall health and relationship status).

#### Initial model: model specification and fit

SEM analysis of the initial, simplest hypothetical model (Figure 1a) indicated statistically significant effect estimates (*p *< .001; Supplementary Material 3) and explained a large proportion of the variance in psychological wellbeing (*R*^2 ^= .84; see Supplementary Material 4 for path diagram and standardised coefficients). However, both local and global fit testing indicated refinements were required to improve model fit. Statistically significant Satorra-Bentler scaled Chi-square test statistics suggested inadequate model fit (*χ*^2^(6, *N = *389) = 27.2, *p *< .001, scaling correction factor = 1.17). All correlation residuals were smaller than .10, indicating good local fit (Supplementary Material 5) (Kline, 2016). Whilst approximately half (53.3%) of the standardised residuals were statistically signficant (*p *< .05), the corresponding correlation residual in each case did not suggest local misfit (>.10), thus local fit was satisfied (Kline, 2016). Whilst the SRMR (.28) and CFI (.974) indicated acceptable model fit, the scaled RMSEA (.095, 90% CI[.063, .130], *p *< .05) and PNFI (.388) did not support a hypothesis of good model fit. The statistical power of the model was marginally lower than the 80% target (power = .793, *df *= 6, *N = *389, RMSEA < .08, *p *< .05). The initial model was therefore rejected, and a more full, complex hypothetical model was tested (Figure 1b).

#### Full model: model specification and fit

**Figure 2.**
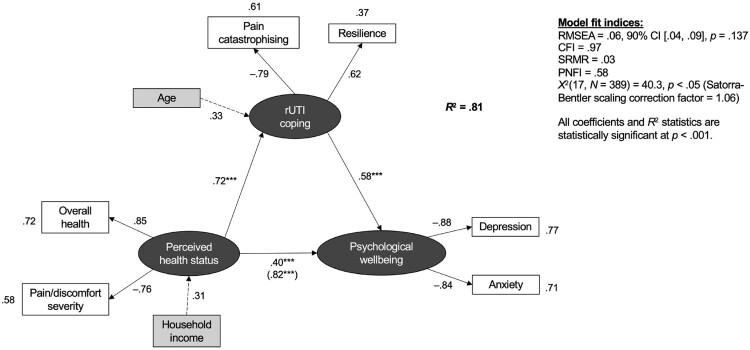
Path diagram illustrating the partially mediated relationship between perceived health status (PHS) and psychological wellbeing in recurrent urinary tract infection (rUTI), with rUTI coping as a mediator. Structural equation modelling (SEM) analysis included three latent variables, presented as dark grey oval shapes: PHS, psychological wellbeing, and rUTI coping. Eight observed variables are presented as rectangles: predictor variables are in white, and covariance variables are in light grey. The values between the latent variables indicate the standardised path (regression) coefficients, each representing a *direct effect* of one variable on another. The coefficient in parentheses between PHS and psychological wellbeing indicates the *total effect* between the two variables. The values between the observed variables and latent variables indicate the standardised factor loadings. The values outside each observed variable are *R*^2^ values, representing the proportion of the variance in the observed variable that is explained by the corresponding latent variable. The higher the percentage of variance of an observed variable that is explained by the latent variable, the better it is at measuring the latent variable. All coefficients and *R*^2^ statistics are statistically significant (*p* < .001). The *R*^2^ value for the overall model is in bold (.81). The direct, indirect, and total effect estimates are reported within the article and summarised in Supplementary Material 3.

Including age and household income as covariance variables resulted in a model with stronger fit, indicating that PHS and rUTI coping explain 81.2% of the variance in psychological wellbeing, representing a large effect size (see Figure 2 for path diagram and standardised coefficients, and Supplementary Material 3 for a summary of model effect estimates) (Kline, 2016; Tabachnick & Fidell, 2012). This full model was named the ‘rUTI Illness Process Model’.

The Satorra-Bentler scaled Chi-square test statistics were statistically significant (*χ*^2^(17, *N = *389) = 40.3, *p *< .01, scaling correction factor = 1.06). However, given the sensitivity of this test to large sample sizes (*N *> 300), it is typical for final fit inferences to be made based on local fit testing and global approximate fit statistics (Kline, 2016; Stone, 2021). All correlation residuals were less than .10 (Supplementary Material 6), indicating that the model appropriately explains the observed association between each pair of variables (Kline, 2016; Stone, 2021). Approximately a quarter (28.6%) of standardised residuals were statistically significant (*p *< .05); all corresponding correlation residuals were smaller than .10, thus local fit was satisfied. All approximate fit indices suggested good global model fit: RMSEA = .066, 90% CI[.041, .092], *p *= .137; SRMR = .035, CFI = .969, PNFI = .580 (Kline, 2016). Further, power analysis indicated the model is highly capable of detecting a model with good approximate fit in the population (power = .996, *df *= 17, *N = *389, RMSEA < .08, *p *< .05).

#### Full model: model effect estimates and interpretation

Overall, demonstrated by the full model, the rUTI Illness Process Model (see Figure 2), it can be concluded that the relationship between PHS and psychological wellbeing is statistically significantly partially mediated by rUTI coping, after controlling for the impact of household income and age on PHS and rUTI coping, respectively (*p *< .001).

As hypothesised, both PHS and rUTI coping demonstrated positive direct effects on psychological wellbeing (.40 and .58, respectively; both *p *< .001). Statistical comparison of standardised path coefficients indicated that PHS had a considerably stronger direct effect on rUTI coping than on psychological wellbeing (.72 and .40, respectively; *p *< .001). Similarly, rUTI coping had a moderately stronger direct effect on psychological wellbeing than PHS (.58 and .40, respectively; *p *< .001). Also as hypothesised, the indirect effect of PHS on psychological wellbeing operating via rUTI coping skills, while controlling for the direct effect of PHS on wellbeing, was moderate and statistically significantly different from zero (.42, 90% CI[.27, .56], *p *< .001). This resulted in a strong total effect of PHS on psychological wellbeing (.82, *p *< .001). Thus, rUTI coping skills, indicated by pain catastrophising tendencies and resilience, contribute to a large proportion of the positive relationship between PHS and psychological wellbeing in rUTI.

All observed variables predicted their related latent variables as hypothesised with a large effect size (*p *< .001; Figure 2). Overall health positively predicts PHS, whereby greater overall health is associated with greater PHS. Conversely, pain/discomfort severity negatively predicts PHS, so greater pain levels implicate poorer PHS. Both depression and anxiety negatively predict psychological wellbeing. Finally, whilst rUTI coping is positively predicted by resilience, it is negatively predicted by pain catastrophising. Thus, resilience strengthens and protects the ability to cope, whilst greater catastrophising weakens this ability.

Age was found to moderately positively predict rUTI coping (*p *< .001), suggesting that older adults are likely to exhibit stronger coping strategies, including higher resilience and lower pain catastrophising. Further, household income was a moderate positive predictor of PHS (*p *< .001), indicating that people of higher socioeconomic status (SES) are more likely to report better health and pain outcomes.

## Discussion

This study established a novel, dynamic model that characterises the unique illness perceptions and metacognitive strategies held by people living with recurrent urinary tract infection (rUTI): the rUTI Illness Process Model. The relationship between perceived health status and psychological wellbeing was partially mediated by coping, yet this relationship was validated after controlling for the influence of age and household income. Coping skills were found to play an essential role in strengthening and promoting the positive influence of perceived health in rUTI, facilitating psychological adjustment to living with rUTI and QoL. A critical combination of higher resilience and lower pain catastrophising enhances the ability to adjust to poor health, thus protecting psychological wellbeing. The highly individuated nature of living with rUTI was illustrated, with participants exhibiting high variability in all observed variables. This study is the first to evaluate and model the interplay between rUTI illness perceptions and psychological wellbeing, adding new insights through assessment of sociodemographic variables such as age and SES. A uniquely vulnerable patient phenotype emerges from this research, with patients who are younger and/or of lower SES at greater risk of poorer rUTI health outcomes and psychological wellbeing.

Whilst the current findings support the patterns identified in some related urological patient populations, including IC/BPS and CP/CPPS, this is the first study to specifically examine and characterise the inter-relationships between perceived health status, psychological wellbeing, and coping in rUTI, and to additionally consider socioeconomic factors such as age and SES. People living with rUTI face distinct challenges due to factors such as inadequate diagnostic approaches, an overreliance upon antimicrobial therapies despite increasing antimicrobial resistance, and high levels of healthcare disillusionment (Brubaker et al., 2023; Gleicher et al., 2023; Haley & Luke, 2024; Harding et al., 2022; Hearn et al., 2018; Maxwell et al., 2023; Pickard et al., 2018; Sanyaolu et al., 2023; Sosland & Stewart, 2021). This novel study provides critical validation by demonstrating a rUTI-specific model of the psychological illness processes experienced by this distinctive patient population, pushing beyond existing knowledge and perceptions of chronic pain experiences more broadly.

Overall, these findings address the urgent need for future rUTI medical and psychosocial interventions to incorporate a more individualised, patient-centred approach, fostering improved patient outcomes and reduced psychological distress and burden. Further, these findings will inform the development of new rUTI-specific psychological interventions aimed at improving rUTI-related QoL and coping, which currently do not exist. Coping factors such as resilience and pain catastrophising should be measured within standard rUTI management, providing clinicians with vital insights into these key indicators of rUTI wellbeing (Campbell-Sills & Stein, 2007; Sullivan et al., 1995). Relevant standardised patient-reported outcome measures (PROMs), such as the PCS and CD-RISC-10, could be incorporated into patient assessment and monitoring to build a broader picture of patient wellbeing, and to establish targets for intervention or further exploration via patient-clinician discussion. In addition to patient monitoring, this study established that rUTI interventions specifically aimed at actively building resilience and reducing pain catastrophising, to improve psychological wellbeing and overall QoL, are essential within clinical management. Close consideration of how people living with rUTI perceive their symptoms, pain severity, and overall health is also crucial, given the predictive value of this perception on psychological wellbeing and QoL. Application of rUTI-specific PROMs, such as the Recurrent UTI Symptom Scale (RUTISS) and the Recurrent UTI Impact Questionnaire (RUTIIQ) (Newlands et al., 2023; Newlands et al., 2024), is recommended to facilitate a standardised approach to exploration and assessment of the rUTI patient perspective of symptoms, pain, and QoL impact.

Further, the rUTI Illness Process Model dynamically represents how key sociodemographic characteristics such as age and SES must not be overlooked. Patient sub-populations, demonstrated by group differences in rUTI coping skills and health outcomes, are at greater risk of more severe psychosocial impact, highlighting the requirement to tailor rUTI management and care. Healthcare professionals should actively consider socioeconomic factors within rUTI management, identifying those most at risk of poorer psychological wellbeing and health outcomes. The rUTI Illness Process Model ultimately provides vital insights for informing new rUTI-specific health psychology interventions through selection of strategic variables and outcome measures. Novel interventions could focus on resilience, pain catastrophising and coping strategies, stress reduction and mindfulness-based techniques, patient-clinician communication, shared decision-making, acceptance and commitment therapeutic approaches, and psychoeducation.

Whilst this study demonstrates important new insights into rUTI illness processes, certain limitations should be addressed. Although rUTI is significantly more prevalent in females (Foxman, 2014; Zeng et al., 2022), gender differences in the patient experience may occur. Additionally, it is acknowledged that most participants were Caucasian, English-speaking, and residing in high-income countries. Financial status was generally high, with approximately one third of participants reporting an annual household income of £50,000 or more, beyond the UK’s average of £34,500 in 2022–2023 (Office for National Statistics, 2024). Education levels were also higher than average, with almost three quarters of participants reporting obtaining at least an undergraduate degree, compared with approximately a third within the general populations in the UK and USA (Office for National Statistics, 2022; United States Census Bureau, 2019). Therefore, further exploration of the proposed model with a more sociodemographically diverse sample would help to identify sub-group differences, such as gender-related and cross-cultural similarities and differences, in the role of rUTI coping. Exploration of additional sociodemographic and socioeconomic variables in the context of the proposed model would also provide a more nuanced understanding of the broader rUTI patient population.

It is also important to recognise that perceived health, coping, and psychological wellbeing are complex latent constructs. Whilst the questionnaires employed to represent them are psychometrically robust, it is possible that using alternative or additional measures to assess each latent construct could enhance the accuracy of that assessment. Future research could also seek to examine the possible influence of other factors, such as social support and life satisfaction, on rUTI coping and psychological wellbeing (Katz et al., 2017). Whilst recruitment of participants based on self-report of rUTI symptoms was appropriate, given published recommendations to prioritise patient symptom reporting (Brubaker et al., 2023; Harding et al., 2022; Pickard et al., 2018), and online recruitment facilitated access to the larger, more heterogeneous sample required for SEM analysis, further validation of the rUTI Illness Process Model using an offline, clinic-based sample may be beneficial to reduce the risk of selection bias. Examination of the model performance in conjunction with other clinical information (e.g., rUTI symptom presentation, comorbidities) may provide additional insights. Future research could explore rUTI-specific coping strategies or interventions and consider more innovative ways to distinguish the psychological experience of rUTI from other chronic conditions. Finally, further studies should implement a longitudinal, experimental trial design to validate the model, identifying coping strategies that may support the model conclusions and inform biopsychosocial intervention development.

## Conclusion

This study is the first to evaluate and model the mediating role of coping in the relationship between perceived health status and psychological wellbeing in recurrent urinary tract infection (rUTI), a common urological condition associated with unique physical and psychological challenges. This study provides critical validation of a new model of the psychological illness processes experienced by this distinctive patient population, pushing beyond existing knowledge and perceptions of chronic pain experiences more broadly. The proposed model established that a combination of high resilience and low pain catastrophising strengthens the ability to adjust to poor health, thus protecting psychological wellbeing, and crucially that individual sociodemographic factors must not be overlooked in rUTI care, with patients who are younger and/or of lower socioeconomic status found to exhibit the greatest risk for poor rUTI health outcomes and psychological wellbeing. With these new, vital insights into rUTI illness processes and perceptions, rUTI management and care can and must become more patient-centred and individualised. The rUTI Illness Process Model and its identified inter-relationships between perceived health status, psychological wellbeing, and rUTI coping can inform the development of the first rUTI-specific psychological interventions, ultimately enhancing patient outcomes and health-related quality of life.

## Supplementary Material

Supplemental Material

Supplemental Material

Supplemental Material

Supplemental Material

Supplemental Material

Supplemental Material

## Data Availability

The datasets used and analysed during the current study are stored in the Open Science Framework data repository 10.17605/OSF.IO/WN4DM.

## Abbreviations

**Abbreviations:** CD-RISC-10: Connor Davidson Resilience Scale–10; CFI: comparative fit index; CP/CPPS: chronic prostatitis/chronic pelvic pain syndrome; EQ-5D-5L: EuroQol 5-dimension 5-level questionnaire; GAD-7: Generalised Anxiety Disorder–7; IC/PBS: interstitial cystitis/bladder pain syndrome; PCS: Pain Catastrophising Scale; PHQ-9: Patient Health Questionnaire–9; PNFI: parsimonious normed fit index; QoL: quality of life; RMSEA: root mean square error of approximation; rUTI: recurrent urinary tract infection; SES: socioeconomic status; SRMR: standardised root mean square residual; UTI: urinary tract infection; VAS: visual analogue scale

## Open scholarship

This article has earned the Center for Open Science badges for Open Data and Open Materials through Open Practices Disclosure. The data and materials are openly accessible at https://doi.org/10.17605/OSF.IO/WN4DM and https://doi.org/10.17605/OSF.IO/WN4DM.

## References

[CIT0001] Bonkat, G., Bartoletti, R., Bruyére, F., Cai, T., Geerlings, S. E., Köves, B., Schubert, S., & Wagenlehner, F. (2020). EAU guidelines on urological infections 2020 (*European Association of Urology Guidelines. 2020 Edition.* (Vol. presented at the EAU Annual Congress Amsterdam 2020). European Association of Urology Guidelines Office. http://uroweb.org/guideline/urological-infections/

[CIT0002] Brubaker, L., Chai, T. C., Horsley, H., Khasriya, R., Moreland, R. B., & Wolfe, A. J. (2023). Tarnished gold—the “standard” urine culture: Reassessing the characteristics of a criterion standard for detecting urinary microbes. *Frontiers in Urology*, *3*, 10.3389/fruro.2023.1206046 PMC12327316 40778073

[CIT0003] Campbell-Sills, L., & Stein, M. B. (2007). Psychometric analysis and refinement of the Connor–Davidson Resilience Scale (CD-RISC): Validation of a 10-item measure of resilience. *Journal of Traumatic Stress*, *20*(6), 1019–1028. 10.1002/jts.20271 18157881

[CIT0004] Chng, Z., Yeo, J. J., & Joshi, A. (2023). Resilience as a protective factor in face of pain symptomatology, disability and psychological outcomes in adult chronic pain populations: A scoping review. *Scandinavian Journal of Pain*, *23*(2), 228–250. 10.1515/sjpain-2021-0190 35946872

[CIT0005] Ciani, O., Grassi, D., & Tarricone, R. (2013). An economic perspective on urinary tract infection: The “costs of resignation”. *Clinical Drug Investigation*, *33*(4), 255–261. 10.1007/s40261-013-0069-x 23475540

[CIT0006] Colgan, R., Keating, K., & Dougouih, M. (2004). Survey of symptom burden in women with uncomplicated urinary tract infections. *Clinical Drug Investigation*, *24*(1), 55–60. 10.2165/00044011-200424010-00007 17516691

[CIT0007] Foxman, B. (2014). Urinary tract infection syndromes: Occurrence, recurrence, bacteriology, risk factors, and disease burden. *Infectious Disease Clinics of North America*, *28*(1), 1–13. 10.1016/j.idc.2013.09.003 24484571

[CIT0008] François, M., Hanslik, T., Dervaux, B., Le Strat, Y., Souty, C., Vaux, S., Maugat, S., Rondet, C., Sarazin, M., Heym, B., Coignard, B., & Rossignol, L. (2016). The economic burden of urinary tract infections in women visiting general practices in France: A cross-sectional survey. *BMC Health Services Research*, *16*(1), 365. 10.1186/s12913-016-1620-2 27507292 PMC4977873

[CIT0009] Giannantoni, A., Gubbiotti, M., Balzarro, M., & Rubilotta, E. (2021). Resilience in the face of pelvic pain: A pilot study in males and females affected by urologic chronic pelvic pain. *Neurourology and Urodynamics*, *40*(4), 1011–1020. 10.1002/nau.24659 33764614 PMC8252554

[CIT0010] Gleicher, S., Sebesta, E. M., Kaufman, M. R., Dmochowski, R. R., & Reynolds, W. S. (2023). Recurrent urinary tract infection management and prevention techniques among a population-based cohort of women. *Neurourology and Urodynamics*, 10.1002/nau.25281 PMC13169508 37670465

[CIT0011] Haley, E., & Luke, N. (2024). From awareness to action: Pioneering solutions for women’s UTI challenges in the era of precision medicine. *International Journal of Women’s Health*, *16*(null), 1595–1605. 10.2147/IJWH.S477476 PMC11446210 39359902

[CIT0012] Harding, C., Mossop, H., Homer, T., Chadwick, T., King, W., Carnell, S., Lecouturier, J., Abouhajar, A., Vale, L., Watson, G., Forbes, R., Currer, S., Pickard, R., Eardley, I., Pearce, I., Thiruchelvam, N., Guerrero, K., Walton, K., Hussain, Z., … Ali, A. (2022). Alternative to prophylactic antibiotics for the treatment of recurrent urinary tract infections in women: Multicentre, open label, randomised, non-inferiority trial. *BMJ*, *376*, e068229. 10.1136/bmj-2021-0068229 35264408 PMC8905684

[CIT0013] Hays, R. D., Revicki, D., & Coyne, K. S. (2005). Application of structural equation modeling to health outcomes research. *Evaluation & the Health Professions*, *28*(3), 295–309. 10.1177/0163278705278277 16123259

[CIT0014] Hearn, J. H., Selvarajah, S., Kennedy, P., & Taylor, J. (2018). Stigma and self-management: An interpretative phenomenological analysis of the impact of chronic recurrent urinary tract infections after spinal cord injury. *Spinal Cord Series and Cases*, *4*(1), 10.1038/s41394-018-0042-2 PMC5809365 29449969

[CIT0015] Hedelin, H. (2012). The chronic prostatitis/chronic pelvic pain syndrome and pain catastrophizing: A vicious combination. *Scandinavian Journal of Urology and Nephrology*, *46*(4), 273–278. 10.3109/00365599.2012.669403 22452520

[CIT0016] Herdman, M., Gudex, C., Lloyd, A., Janssen, M., Kind, P., Parkin, D., Bonsel, G., & Badia, X. (2011). Development and preliminary testing of the new five-level version of EQ-5D (EQ-5D-5L). *Quality of Life Research*, *20*(10), 1727–1736. 10.1007/s11136-011-9903-x 21479777 PMC3220807

[CIT0017] Izett-Kay, M., Barker, K. L., McNiven, A., & Toye, F. (2022). Experiences of urinary tract infection: A systematic review and meta-ethnography. *Neurourology and Urodynamics*, *41*(3), 724–739. 10.1002/nau.24884 35114012

[CIT0018] Jones, L. F., Cooper, E., Joseph, A., Allison, R., Gold, N., Donald, I., & McNulty, C. (2020). Development of an information leaflet and diagnostic flow chart to improve the management of urinary tract infections in older adults: A qualitative study using the theoretical domains framework. *BJGP Open*, *4*(3), bjgpopen20X101044. 10.3399/bjgpopen20X101044 PMC7465577 32576575

[CIT0019] Kanter, G., Komesu, Y. M., Qaedan, F., Jeppson, P. C., Dunivan, G. C., Cichowski, S. B., & Rogers, R. G. (2016). Mindfulness-based stress reduction as a novel treatment for interstitial cystitis/bladder pain syndrome: A randomized controlled trial. *International Urogynecology Journal*, *27*(11), 1705–1711. 10.1007/s00192-016-3022-8 27116196 PMC5067184

[CIT0020] Katz, L., Tripp, D. A., Carr, L. K., Mayer, R., Moldwin, R. M., & Nickel, J. C. (2017). Understanding pain and coping in women with interstitial cystitis/bladder pain syndrome. *BJU International*, *120*(2), 286–292. 10.1111/bju.13874 28386966

[CIT0021] Kline, R. B. (2016). *Principles and practice of structural equation modeling* (4th ed.). Guilford Press.

[CIT0022] Kroenke, K., Spitzer, R. L., & Williams, J. B. (2001). The PHQ-9: Validity of a brief depression severity measure. *Journal of General Internal Medicine*, *16*(9), 606–613. 10.1046/j.1525-1497.2001.016009606.x 11556941 PMC1495268

[CIT0023] Lee, M.-H., Wu, H.-C., Tseng, C.-M., Ko, T.-L., Weng, T.-J., & Chen, Y.-F. (2018). Health education and symptom flare management using a video-based m-health system for caring women with IC/BPS. *Urology*, *119*, 62–69. 10.1016/j.urology.2018.05.027 29894774

[CIT0024] Maxwell, K., Roberts, L., Kramer, M., Price, J. L., Newlands, A. F., & Finlay, K. A. (2023). Psychosocial burden and healthcare disillusionment in recurrent UTI: A large-scale international survey of patient perspectives. *Frontiers in Urology*, *3*, 1264299. 10.3389/fruro.2023.1264299 PMC12327290 40778078

[CIT0025] Medina, M., & Castillo-Pino, E. (2019). An introduction to the epidemiology and burden of urinary tract infections. *Therapeutic Advances in Urology*, *11*, 175628721983217. 10.1177/1756287219832172 PMC6502976 31105774

[CIT0026] Metwally, A., Abdelaziz, A., Ghalwash, M., & Mohamed, A. (2021). Effect of self-care practice health educational program for patients on urinary tract infection recurrence. *Tanta Scientific Nursing Journal*, *23*(4), 134–159. 10.21608/tsnj.2021.208722

[CIT0027] Mirowsky, J. (2017). *Education, socioeconomic status, and health (Education, social status, and health)*. Routledge.

[CIT0028] Morrison, T., Morrison, M., & McCutcheon, J. (2017). Best practice recommendations for using structural equation modelling in psychological research. *Psychology (Savannah, GA)*, *8*, 1326–1341. 10.4236/psych.2017.89086

[CIT0029] Naber, K. G., Tirán-Saucedo, J., & Wagenlehner, F. M. E. (2022). Psychosocial burden of recurrent uncomplicated urinary tract infections. *GMS Infectious Disease*, *10*, Doc01. 10.3205/id000078 PMC9006425 35463815

[CIT0030] Naliboff, B. D., Stephens, A. J., Afari, N., Lai, H., Krieger, J. N., Hong, B., Lutgendorf, S., Strachan, E., & Williams, D. (2015). Widespread psychosocial difficulties in men and women with urologic chronic pelvic pain syndromes: Case-control findings from the multidisciplinary approach to the study of chronic pelvic pain research network. *Urology*, *85*(6), 1319–1327. 10.1016/j.urology.2015.02.047 26099876 PMC4479402

[CIT0031] Newlands, A. F., Kramer, M., Roberts, L., Maxwell, K., Price, J. L., & Finlay, K. A. (2024). Confirmatory structural validation and refinement of the Recurrent Urinary Tract Infection Symptom Scale. *BJUI Compass*, *5*(2), 240–252. 10.1002/bco2.297 38371201 PMC10869661

[CIT0032] Newlands, A. F., Roberts, L., Maxwell, K., Kramer, M., Price, J. L., & Finlay, K. A. (2023). Development and psychometric validation of a patient-reported outcome measure of recurrent urinary tract infection impact: The Recurrent UTI Impact Questionnaire. *Quality of Life Research*, *32*(6), 1745–1758. 10.1007/s11136-023-03348-7 36740638 PMC10172217

[CIT0033] Noy, C. (2008). Sampling knowledge: The hermeneutics of snowball sampling in qualitative research. *International Journal of Social Research Methodology*, *11*(4), 327–344. 10.1080/13645570701401305

[CIT0034] Office for National Statistics. (2022). *Education, England and wales: Census* 2021 (https://www.ons.gov.uk/peoplepopulationandcommunity/educationandchildcare/bulletins/educationenglandandwales/census2021).

[CIT0035] Office for National Statistics. (2024). *Average household income, UK: Financial year ending* 2023 (https://www.ons.gov.uk/peoplepopulationandcommunity/personalandhouseholdfinances/incomeandwealth/bulletins/householddisposableincomeandinequality/financialyearending2023).

[CIT0036] Ong, A. D., Zautra, A. J., & Reid, M. C. (2010). Psychological resilience predicts decreases in pain catastrophizing through positive emotions. *Psychology and Aging*, *25*(3), 516. 10.1037/a0019384 20853962 PMC3626095

[CIT0037] Pickard, R., Chadwick, T., Oluboyede, Y., Brennand, C., von Wilamowitz-Moellendorff, A., McClurg, D., Wilkinson, J., Ternent, L., Fisher, H., Walton, K., McColl, E., Vale, L., Wood, R., Abdel-Fattah, M., Hilton, P., Fader, M., Harrison, S., Larcombe, J., Little, P., … Thiruchelvam, N. (2018). Continuous low-dose antibiotic prophylaxis to prevent urinary tract infection in adults who perform clean intermittent self-catheterisation: The AnTIC RCT. *Health Technology Assessment*, *22*(24), 1–102. 10.3310/hta22240 PMC5971229 29766842

[CIT0038] Ramírez-Maestre, C., & Esteve, R. (2013). Disposition and adjustment to chronic pain. *Current Pain and Headache Reports*, *17*(3), 312. 10.1007/s11916-012-0312-9 23338768

[CIT0039] Renard, J., Ballarini, S., Mascarenhas, T., Zahran, M., Quimper, E., Choucair, J., & Iselin, C. E. (2015). Recurrent lower urinary tract infections have a detrimental effect on patient quality of life: A prospective, observational study. *Infectious Diseases and Therapy*, *4*(1), 125–135. 10.1007/s40121-014-0054-6 PMC4363217 25519161

[CIT0040] Sanyaolu, L. N., Hayes, C. V., Lecky, D. M., Ahmed, H., Cannings-John, R., Weightman, A., Edwards, A., & Wood, F. (2023). Patients’ and healthcare professionals’ experiences and views of recurrent urinary tract infections in women: Qualitative evidence synthesis and meta-ethnography. *Antibiotics*, *12*(3), 434. 10.3390/antibiotics12030434 36978301 PMC10044648

[CIT0041] Schumacker, R. E., & Lomax, R. G. (2010). *A beginner’s guide to structural equation modeling* (3rd ed.). Routledge.

[CIT0042] Smith, B. W., Epstein, E. M., Ortiz, J. A., Christopher, P. J., & Tooley, E. M. (2013). The foundations of resilience: What are the critical resources for bouncing back from stress? In S. Prince-Embury, & D. H. Saklofske (Eds.), *Resilience in children, adolescents, and adults: Translating research into practice* (pp. 167–187). Springer.

[CIT0043] Soper, D. S. (2022). *A-priori sample size calculator for structural equation models*. https://www.danielsoper.com/statcalc

[CIT0044] Soriano, A., Allen, A., Malykhina, A. P., Andy, U., Harvie, H., & Arya, L. (2021). Relationship of pain catastrophizing with urinary biomarkers in women with bladder pain syndrome. *Female Pelvic Medicine & Reconstructive Surgery*, *27*(12), 746–752. 10.1097/spv.0000000000001041 33787562 PMC8449794

[CIT0045] Sosland, R., & Stewart, J. N. (2021). Management of recurrent urinary tract infections in women: How providers can improve the patient experience. *Urology*, *151*, 8–12. 10.1016/j.urology.2020.06.059 32673677

[CIT0046] Spitzer, R. L., Kroenke, K., Williams, J. B. W., & Löwe, B. (2006). A brief measure for assessing generalized anxiety disorder: The GAD-7. *Archives of Internal Medicine*, *166*(10), 1092. 10.1001/archinte.166.10.1092 16717171

[CIT0047] Stone, B. M. (2021). The ethical use of fit indices in structural equation modeling: Recommendations for psychologists. *Frontiers in Psychology*, *12*, 10.3389/fpsyg.2021.783226 PMC8650002 34887821

[CIT0048] Sullivan, M. J. L., Bishop, S. R., & Pivik, J. (1995). The Pain Catastrophizing Scale: Development and validation. *Psychological Assessment*, *7*(4), 524–532. 10.1037/1040-3590.7.4.524

[CIT0049] Tabachnick, B. G., & Fidell, L. S. (2012). *Using multivariate statistics* (6th ed.). Pearson.

[CIT0050] United States Census Bureau. (2019). *Educational attainment in the United States:* 2018 (https://www.census.gov/data/tables/2018/demo/education-attainment/cps-detailed-tables.html)

[CIT0051] Wagenlehner, F., Wullt, B., Ballarini, S., Zingg, D., & Naber, K. G. (2018). Social and economic burden of recurrent urinary tract infections and quality of life: A patient web-based study (GESPRIT). *Expert Review of Pharmacoeconomics & Outcomes Research*, *18*(1), 107–117. 10.1080/14737167.2017.1359543 28737469

[CIT0052] Zeng, Z., Zhan, J., Zhang, K., Chen, H., & Cheng, S. (2022). Global, regional, and national burden of urinary tract infections from 1990 to 2019: An analysis of the global burden of disease study 2019. *World Journal of Urology*, *40*(3), 755–763. 10.1007/s00345-021-03913-0 35066637

[CIT0053] Ziadni, M. S., Sturgeon, J. A., & Darnall, B. D. (2018). The relationship between negative metacognitive thoughts, pain catastrophizing and adjustment to chronic pain. *European Journal of Pain*, *22*(4), 756–762. 10.1002/ejp.1160 29214679 PMC5854507

