# Supplementary material for: The mediating role of coping in the relationship between perceived health and psychological wellbeing in recurrent urinary tract infection: the rUTI Illness Process Model
Source: Health Psychol Behav Med. 2024 Nov 3;12(1):2420806. doi: 10.1080/21642850.2024.2420806 (PMC11536654; doi:10.1080/21642850.2024.2420806)
Supplement: Supplemental Material [file RHPB_A_2420806_SM2429.docx]

**Supplementary Material 1.** Full country of residence frequencies

| Country | *n* | % |
| --- | --- | --- |
| United Kingdom | 153 | 39.3 |
| United States | 147 | 37.8 |
| Canada | 26 | 6.68 |
| Australia | 8 | 2.06 |
| Ireland | 5 | 1.29 |
| Greece | 4 | 1.03 |
| India | 4 | 1.03 |
| Spain | 4 | 1.03 |
| Germany | 3 | .77 |
| Israel | 3 | .77 |
| South Africa | 3 | .77 |
| France | 2 | .51 |
| Norway | 2 | .51 |
| Sweden | 2 | .51 |
| Angola | 1 | .26 |
| Argentina | 1 | .26 |
| Austria | 1 | .26 |
| The Bahamas | 1 | .26 |
| Belgium | 1 | .26 |
| Croatia | 1 | .26 |
| Czech Republic | 1 | .26 |
| Denmark | 1 | .26 |
| Finland | 1 | .26 |
| Iceland | 1 | .26 |
| Italy | 1 | .26 |
| Jersey | 1 | .26 |
| Malawi | 1 | .26 |
| Mexico | 1 | .26 |
| Netherlands | 1 | .26 |
| New Zealand | 1 | .26 |
| Nigeria | 1 | .26 |
| Romania | 1 | .26 |
| Serbia | 1 | .26 |
| Slovakia | 1 | .26 |
| Thailand | 1 | .26 |
| Turkey | 1 | .26 |
| Ukraine | 1 | .26 |

*Note.* *N* = 389. In total, participants came from 37 countries.
