# Supplementary material for: The mediating role of coping in the relationship between perceived health and psychological wellbeing in recurrent urinary tract infection: the rUTI Illness Process Model
Source: Health Psychol Behav Med. 2024 Nov 3;12(1):2420806. doi: 10.1080/21642850.2024.2420806 (PMC11536654; doi:10.1080/21642850.2024.2420806)
Supplement: Supplemental Material [file RHPB_A_2420806_SM2426.docx]

**Supplementary Material 2:** This file provides further details on the data handling strategy implemented to collect participant data (with a sampling flow diagram), prepare and analyse the data with structural equation modelling, as well as explore the descriptive statistics. References are provided at the bottom of the file.

| **Data preparation and testing statistical assumptions** |  | The full dataset was screened for **missing data** and **ineligible participants** based on the specified inclusion and exclusion criteria, resulting in a final sample of 389 participants *(see below for sampling flow diagram)*.  Data for each standardised questionnaire was **scored** according to their scoring algorithm [1-5]. | | |
| --- | --- | --- | --- | --- |
|  |  |  |  |  |
|  |  | The assumption of multivariate normality was checked by computing **Mardia’s multivariate skewness** and **kurtosis coefficients** [6, 7].  Both coefficients were statistically significant (*p<*.05), thus a **robust maximum likelihood estimation** was applied to correct for non-normality, computing robust standard errors and Satorra-Bentler scaled test statistics [6-9]. | | |
|  |  |  |  |  |
| **Model specification** |  | Structural equation model (SEM) analysis was conducted in R using the ‘**lavaan**’ package [6]. In accordance with SEM best practice and the parsimony principle [10], the simplest model possible was specified as the initial model. | | |
|  |  |  |  |  |
|  |  | **Initial model (Figure 1a)** |  | The initially tested hypothetical model specified three latent variables (perceived health status: PHS, rUTI coping, and psychological wellbeing) and six observed variables (see Figure 1a).  PHS was manifested by overall health (EQ-5D VAS score) and pain/discomfort severity (EQ-5D pain/discomfort item). rUTI coping was estimated by pain catastrophising (PCS total score) and resilience (CD-RISC-10 total score). Psychological wellbeing was manifested by depression (PHQ-9 total score) and anxiety (GAD-7 total score).  Regression relationships were specified between each of the three latent variables. |
|  |  |  |  |  |
|  |  | **Full model (Figure 1b)** |  | Based on research indicating that household income positively predicts health [11], and that age positively predicts coping factors such as resilience [12], these sociodemographic factors were explored as covariance variables to strengthen the initial model fit and statistical significance (see Figure 1b) [10]. |
|  |  |  |  |  |
| **Model fit evaluation** |  | **The following strategy was applied to evaluate each model separately (Fig 1a and Fig 1b).** | | |
|  |  |  |  |  |
|  |  | **Chi-square statistics** |  | The model fit indices and parameters were evaluated according to Kline’s recommended approach [6, 7, 10]. **Satorra-Bentler scaled Chi-square test statistics** were examined, interpreting non-significant results as suggestive of good model fit (*p*>.05) [9, 10, 13].  Given the sensitivity of this exact-fit test to large sample sizes (*N>*300), it is typical for final fit inferences to be made based on local fit testing, including examination of residuals, and global approximate fit statistics [10, 14]. |
|  |  |  |  |  |
|  |  | **Local fit testing** |  | **Correlation residuals** smaller than .10 suggest good local fit, indicating that the model appropriately explains the observed association between each pair of variables [10, 14]. **Standardised residuals** were interpreted as *Z*-scores following Kline [10], with statistically significant values (*p<*.05) suggesting possible local fit issues. However, this test is sensitive to larger sample sizes [10]. Therefore, in the case of significant standardised residuals, the model was retained unless the corresponding correlation residual also indicated local misfit (>.10) [10]. |
|  |  |  |  |  |
|  |  | **Global fit testing** |  | The following **approximate fit indices** were assessed: root mean square error of approximation (**RMSEA**), standardised root mean square residual (**SRMR**), comparative fit index (**CFI**), and parsimonious normed fit index (**PNFI**).  It was specified a priori that the following thresholds would indicate good fit: scaled RMSEA below .08, with its entire 90% confidence interval below .10 (*p*>.05); SRMR below .08; CFI above .95; and PNFI greater than .50 [10]. |
|  |  |  |  |  |
|  |  | **Statistical power testing** |  | The **statistical power** of the model was calculated using ‘semTools’ in R, estimating the probability of detecting a model with good approximate fit in the population (*p*<.05, *N*=389, RMSEA<.08) [10]. A minimum target of 80% power was specified a priori [10]. |
|  |  |  |  |  |
|  |  | **Model effect estimates** |  | The strength of direct and indirect effects was examined by assessing the **standardised path coefficients**, with coefficients closer to +1 representing strong positive relationships and coefficients closer to –1 representing strong negative relationships. Standardised path coefficients facilitate interpretation such that a coefficient of .80 means that when the predictor (e.g., PHS) increases by 1 *SD* unit, the predicted variable (e.g., rUTI coping) increases by .80 *SD* units.  ***R*^2^ values** were computed to determine the predictive values of each observed variable within the model overall, and of each latent variable on each other [7].  Both standardised path coefficients and *R*^2^ values were expected to be statistically significant (*p<*.05) [10].  **Standardised factor loadings** for observed variables on latent variables were examined, with values closer to 1 indicating stronger relationships [7]. |
|  |  |  |  |  |
| **Descriptive statistics** |  | The sample’s descriptive statistics were explored in R, evaluating their **sociodemographic characteristics**, and conducting **multiple regression analyses** to identify any statistically significant group differences (*p*<.05). | | |
|  |  |  |  |  |

**Sampling flow diagram:**

**
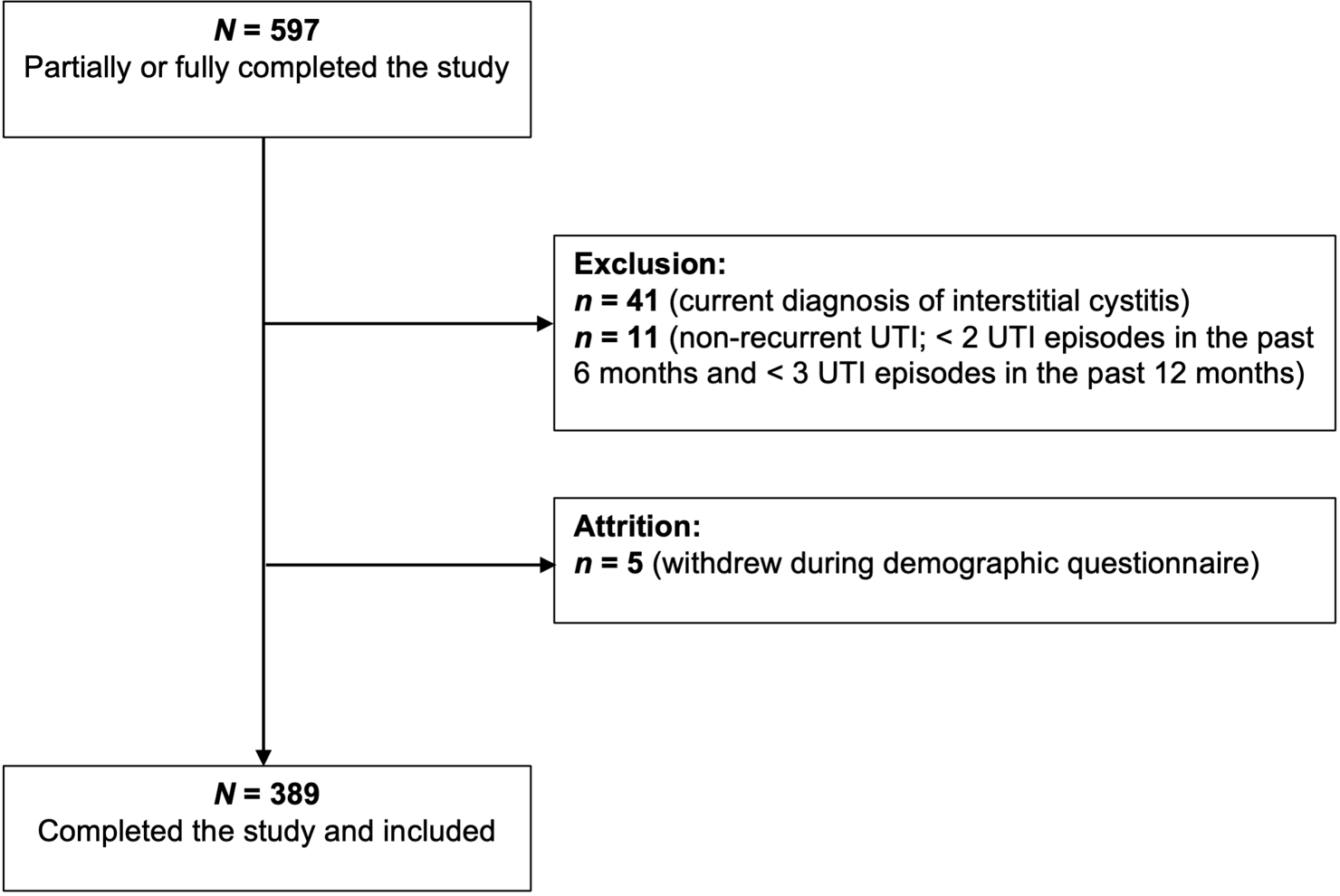
**

**References:**

1. Kroenke K, Spitzer RL, Williams JB. The PHQ-9: Validity of a brief depression severity measure. J Gen Intern Med 2001; 16(9): 606-13.

2. Spitzer RL, Kroenke K, Williams JBW, Löwe B. A brief measure for assessing generalized anxiety disorder: The GAD-7. Arch Intern Med 2006; 166(10): 1092.

3. Herdman M, Gudex C, Lloyd A, et al. Development and preliminary testing of the new five-level version of EQ-5D (EQ-5D-5L). Qual Life Res 2011; 20(10): 1727-36..

4. Campbell-Sills L, Stein MB. Psychometric analysis and refinement of the Connor–Davidson Resilience Scale (CD-RISC): Validation of a 10-item measure of resilience. J Trauma Stress 2007; 20(6): 1019-28.

5. Sullivan MJL, Bishop SR, Pivik J. The Pain Catastrophizing Scale: Development and validation. Psychol Assess 1995; 7: 524-32.

6. Rosseel Y. lavaan: An R package for structural equation modeling. *J Stat Softw* 2012; 48(2): 1-36.

7. Tabachnick BG, Fidell LS. Using multivariate statistics, 6th edn. San Francisco: Pearson, 2012.

8. Finney SJ, DiStefano C. Non-normal and categorical data in structural equation modeling. In Hancock GR, Mueller RD eds, Structural equation modeling: A second course. Information Age Publishing, 2008: 269-314.

9. Satorra A, Bentler PM. Corrections to test statistics and standard errors in covariance structure analysis. In von Eye A, Clogg CC eds, Latent variables analysis: Applications for developmental research. Thousands Oaks: Sage, 1994: 399-419.

10. Kline RB. Principles and practice of structural equation modeling, 4th edn. New York: Guilford Press, 2016.

11. Mirowsky J. Education, socioeconomic status, and health. United Kingdom: Routledge, 2017.

12. Smith BW, Epstein EM, Ortiz JA, Christopher PJ, Tooley EM. The foundations of resilience: What are the critical resources for bouncing back from stress? In Prince-Embury S, Saklofske DH eds, Resilience in children, adolescents, and adults: Translating research into practice. New York: Springer New York, 2013: 167-87.

13. Schumacker RE, Lomax RG. A beginner’s guide to structural equation modeling, 3rd edn. Oxfordshire: Routledge, 2010.

14. Stone BM. The ethical use of fit indices in structural equation modeling: Recommendations for psychologists. Front Psychol 2021; 12..
