# Supplementary material for: The mediating role of coping in the relationship between perceived health and psychological wellbeing in recurrent urinary tract infection: the rUTI Illness Process Model
Source: Health Psychol Behav Med. 2024 Nov 3;12(1):2420806. doi: 10.1080/21642850.2024.2420806 (PMC11536654; doi:10.1080/21642850.2024.2420806)
Supplement: Supplemental Material [file RHPB_A_2420806_SM2421.docx]

**Supplementary Material 3.** Initial and full model effect estimates

| Effects | Variable relation | Standardised path coefficient | *SE* |
| --- | --- | --- | --- |
| Initial model |  |  |  |
| Total | Perceived health status 🡪 psychological wellbeing | .82 | .05 |
| Indirect | Perceived health status 🡪 rUTI coping 🡪 psychological wellbeing | .47 | .08 |
| Direct | Perceived health status 🡪 psychological wellbeing | .33 | .10 |
|  | Perceived health status 🡪 rUTI coping | .74 | .05 |
|  | rUTI coping 🡪 psychological wellbeing | .64 | .10 |
| Full model |  |  |  |
| Total | Perceived health status 🡪 psychological wellbeing | .82 | .05 |
| Indirect | Perceived health status 🡪 rUTI coping 🡪 psychological wellbeing | .42 | .07 |
| Direct | Perceived health status 🡪 psychological wellbeing | .40 | .09 |
|  | Perceived health status 🡪 rUTI coping | .72 | .06 |
|  | rUTI coping 🡪 psychological wellbeing | .58 | .09 |

*Note.* *N* = 389. *SE* = standard error.

All standardised path coefficients are statistically significant (*p* < .001).
