# Supplementary figures and images for: The mediating role of coping in the relationship between perceived health and psychological wellbeing in recurrent urinary tract infection: the rUTI Illness Process Model
Source: Health Psychol Behav Med. 2024 Nov 3;12(1):2420806. doi: 10.1080/21642850.2024.2420806 (PMC11536654; doi:10.1080/21642850.2024.2420806)

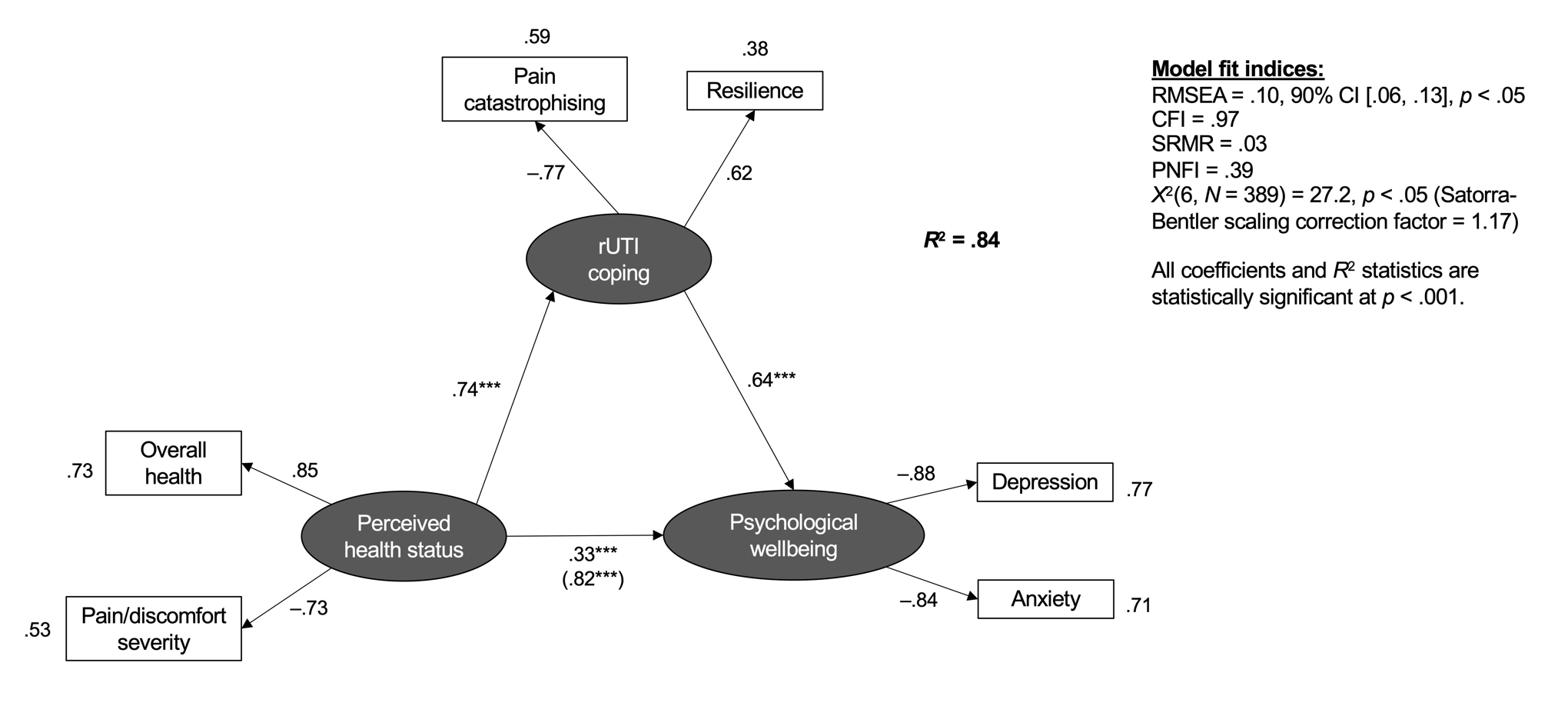

Supplement: Supplemental Material [file RHPB_A_2420806_SM2416.docx]
