# Supplementary material for: The mediating role of coping in the relationship between perceived health and psychological wellbeing in recurrent urinary tract infection: the rUTI Illness Process Model
Source: Health Psychol Behav Med. 2024 Nov 3;12(1):2420806. doi: 10.1080/21642850.2024.2420806 (PMC11536654; doi:10.1080/21642850.2024.2420806)
Supplement: Supplemental Material [file RHPB_A_2420806_SM2415.docx]

**Supplementary Material 5.** Correlation and standardised residuals for the initial model

| Variable | EQ-5D VAS | EQ-5D pain | CD-RISC-10 | PCS | PHQ-9 | GAD-7 | |  |  |  |  |
| --- | --- | --- | --- | --- | --- | --- | --- | --- | --- | --- | --- |
|  | Correlation residuals | | | | | | |  |  |  |  |
| EQ-5D VAS | .000 |  |  |  |  |  | |  |  |  |  |
| EQ-5D pain | .000 | .000 |  |  |  |  | |  |  |  |  |
| CD-RISC-10 | .048 | .035 | .000 |  |  |  | |  |  |  |  |
| PCS | .013 | –.006 | .000 | .000 |  |  | |  |  |  |  |
| PHQ-9 | –.029 | .045 | .028 | –.032 | .000 |  | |  |  |  |  |
| GAD-7 | .045 | –.048 | –.023 | .051 | .000 | .000 | |  |  |  |  |
|  | Standardised residuals | | | | | |  | |  |  |  |
| EQ-5D VAS | .000 |  |  |  |  |  | |  |  |  |  |
| EQ-5D pain | .000 | .000 |  |  |  |  | |  |  |  |  |
| CD-RISC-10 | **2.033** | 1.160 | .000 |  |  |  | |  |  |  |  |
| PCS | .936 | –.271 | .000 | .000 |  |  | |  |  |  |  |
| PHQ-9 | **–2.959** | **2.935** | **2.020** | **–3.368** | .000 |  | |  |  |  |  |
| GAD-7 | **3.754** | **–2.895** | –1.249 | **3.957** | .000 | .000 | |  |  |  |  |

*Note.* *N* = 389. Standardised residuals (*z)* in bold are statistically significant (*p* < .05).

EQ-5D VAS = visual analogue scale evaluating overall health, from the EQ-5D-5L questionnaire (Herdman et al., 2011). PCS = Pain Catastrophizing Scale (Sullivan et al., 1995). CD-RISC-10 = Connor-Davidson Resilience Scale – 10 (Campbell-Sills & Stein, 2007). PHQ-9 = Patient Health Questionnaire – 9 (Kroenke et al., 2001). GAD-7 = Generalized Anxiety Disorder – 7 (Spitzer et al., 2006).
