# Supplementary material for: The mediating role of coping in the relationship between perceived health and psychological wellbeing in recurrent urinary tract infection: the rUTI Illness Process Model
Source: Health Psychol Behav Med. 2024 Nov 3;12(1):2420806. doi: 10.1080/21642850.2024.2420806 (PMC11536654; doi:10.1080/21642850.2024.2420806)
Supplement: Supplemental Material [file RHPB_A_2420806_SM2414.docx]

**Supplementary Material 6.** Correlation and standardised residuals for the full model

| Variable | EQ-5D VAS | EQ-5D pain | CD-RISC-10 | PCS | PHQ-9 | GAD-7 | Income | Age |
| --- | --- | --- | --- | --- | --- | --- | --- | --- |
|  | Correlation residuals | | | | | | | |
| EQ-5D VAS | .000 |  |  |  |  |  |  |  |
| EQ-5D pain | .000 | .000 |  |  |  |  |  |  |
| CD-RISC-10 | .049 | .026 | .000 |  |  |  |  |  |
| PCS | .005 | –.020 | .015 | .000 |  |  |  |  |
| PHQ-9 | –.037 | .021 | –.002 | –.037 | .000 |  |  |  |
| GAD-7 | .051 | –.037 | –.036 | .048 | .000 | .000 |  |  |
| Income | –.024 | –.062 | .053 | .026 | –.007 | .031 | .000 |  |
| Age | –.009 | –.005 | –.052 | –.046 | .089 | –.042 | .082 | .000 |
|  | Standardised residuals | | | | | | | |
| EQ-5D VAS | .000 |  |  |  |  |  |  |  |
| EQ-5D pain | –.053 | .000 |  |  |  |  |  |  |
| CD-RISC-10 | **1.736** | .781 | .000 |  |  |  |  |  |
| PCS | .242 | –.868 | 1.367 | .000 |  |  |  |  |
| PHQ-9 | **–3.391** | 1.475 | –.103 | **–2.840** | .000 |  |  |  |
| GAD-7 | **3.378** | **–2.067** | –1.554 | **3.315** | –.101 | .000 |  |  |
| Income | –1.265 | **–2.133** | 1.055 | .621 | –.206 | .792 | .000 |  |
| Age | –.150 | –.096 | –1.059 | –1.363 | **1.871** | –.965 | 1.354 | .000 |

*Note.* *N* = 389. Standardised residuals (*z)* in bold are statistically significant (*p* < .05).

EQ-5D VAS = visual analogue scale evaluating overall health, from the EQ-5D-5L questionnaire (Herdman et al., 2011). PCS = Pain Catastrophizing Scale (Sullivan et al., 1995). CD-RISC-10 = Connor-Davidson Resilience Scale – 10 (Campbell-Sills & Stein, 2007). PHQ-9 = Patient Health Questionnaire – 9 (Kroenke et al., 2001). GAD-7 = Generalized Anxiety Disorder – 7 (Spitzer et al., 2006). Income = household income (GBP). Age = age in years.
